# 2-Amino-1,3-benzothia­zol-3-ium dihydrogen phosphate

**DOI:** 10.1107/S1600536810025547

**Published:** 2010-07-07

**Authors:** Sabahat Zahra Siddiqui, Usama Waqas, Mehmet Akkurt, Aziz-ur-Rehman, Islam Ullah Khan

**Affiliations:** a Department of Chemistry, Government College University, Lahore 54000, Pakistan; b Department of Physics, Faculty of Arts and Sciences, Erciyes University, 38039 Kayseri, Turkey

## Abstract

The cation of the title compound, C_7_H_7_N_2_S^+^·H_2_PO_4_^−^, is almost planar (r.m.s deviation = 0.017 Å for all non-H atoms). In the crystal structure, the cations and anions are connected by N—H⋯O and O—H⋯O hydrogen bonds, with π–π stacking inter­actions between neighbouring 1,3-thia­zole and benzene rings [centroid–centroid distance = 3.5711 (11) Å], forming a three-dimensional network.

## Related literature

For the structural parameters of some organic dihydrogeno­monophosphates, see: Gholivand *et al.* (2007 ▶); Mrad *et al.* (2009 ▶). For the biological and pharmacological properties of heterocyclic compounds, see: Malik *et al.* (2010 ▶); Sinha & Tiwari (1986 ▶). For the synthesis, see: Thomas *et al.* (2003 ▶).
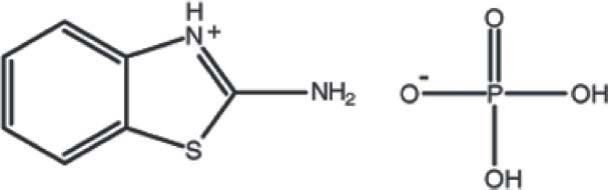


## Experimental

### 

#### Crystal data

C_7_H_7_N_2_S^+^·H_2_PO_4_^−^
*M* *_r_* = 248.20
Monoclinic, 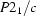

*a* = 12.3915 (4) Å
*b* = 10.1572 (3) Å
*c* = 8.3159 (2) Å
β = 103.775 (1)°
*V* = 1016.56 (5) Å^3^
*Z* = 4
Mo *K*α radiation
μ = 0.47 mm^−1^
*T* = 296 K
0.25 × 0.09 × 0.07 mm

#### Data collection

Bruker APEXII CCD diffractometer
9333 measured reflections
2490 independent reflections
1938 reflections with *I* > 2σ(*I*)
*R*_int_ = 0.033

#### Refinement

*R*[*F* ^2^ > 2σ(*F* ^2^)] = 0.038
*wR*(*F* ^2^) = 0.096
*S* = 1.03
2490 reflections
151 parameters
5 restraints
H atoms treated by a mixture of independent and constrained refinement
Δρ_max_ = 0.31 e Å^−3^
Δρ_min_ = −0.26 e Å^−3^

### 

Data collection: *APEX2* (Bruker, 2007 ▶); cell refinement: *SAINT* (Bruker, 2007 ▶); data reduction: *SAINT*; program(s) used to solve structure: *SHELXS97* (Sheldrick, 2008 ▶); program(s) used to refine structure: *SHELXL97* (Sheldrick, 2008 ▶); molecular graphics: *ORTEP-3 for Windows* (Farrugia, 1997 ▶); software used to prepare material for publication: *WinGX* (Farrugia, 1999 ▶) and *PLATON* (Spek, 2009 ▶).

## Supplementary Material

Crystal structure: contains datablocks global, I. DOI: 10.1107/S1600536810025547/bt5282sup1.cif

Structure factors: contains datablocks I. DOI: 10.1107/S1600536810025547/bt5282Isup2.hkl

Additional supplementary materials:  crystallographic information; 3D view; checkCIF report

## Figures and Tables

**Table 1:** Hydrogen-bond geometry (Å, °)

*D*—H⋯*A*	*D*—H	H⋯*A*	*D*⋯*A*	*D*—H⋯*A*
N1—H1⋯O2^i^	0.873 (16)	1.800 (16)	2.6693 (18)	174 (3)
N2—H6⋯O3^ii^	0.86 (2)	2.39 (2)	3.138 (2)	147 (2)
N2—H6⋯O4^ii^	0.86 (2)	2.31 (2)	3.076 (2)	149.3 (19)
N2—H7⋯O3^i^	0.862 (18)	2.018 (18)	2.875 (2)	172 (2)
O1—H8⋯O2^iii^	0.809 (19)	1.795 (19)	2.6017 (18)	175 (3)
O4—H9⋯O3^iv^	0.801 (17)	1.765 (17)	2.5654 (18)	178 (3)

Symmetry codes: (i) 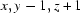; (ii) 
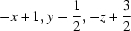
; (iii) 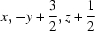; (iv) 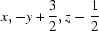.

**Fig. 1.**
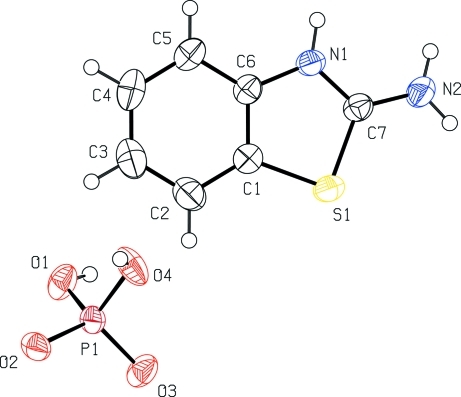
A view of the title molecule. Displacement ellipsoids are drawn at the 50% probability level.

**Fig. 2.**
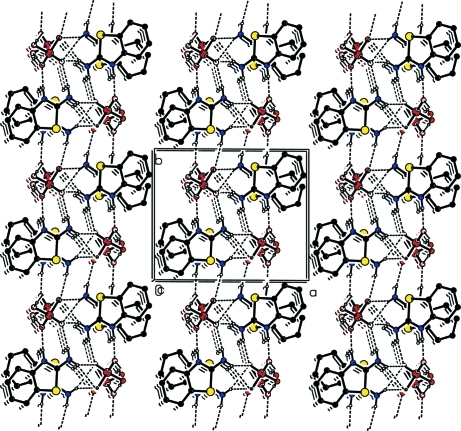
Packing diagram viewed down the c axis. Only H atoms involved in hydrogen bonding are shown.

## supplementary crystallographic information

### Comment

In recent years analogues and derivatives of heterocyclic compounds have attracted strong interest due to their useful biological and pharmacological properties. The substituted benzothiazole derivatives have been reported to possess good antibacterial and antifungal activities. Several of its metal complexes have also displayed potent anti-neoplastic, anti-viral and anti-tumour activities (Malik *et al.*, 2010; Sinha & Tiwari, 1986). In the present paper, the structure of 2-amino-1,3-benzothiazol-3-ium dihydrogen phosphate has been determined as part of a research program involving the synthesis and biological evaluation of sulfur containing compounds.

In the cation of the title compound (I), (Fig. 1), the 1,3-benzothiazol-3-ium ring system (S1/N1/C1–C7) is almost planar (r.m.s deviation = 0.002 Å). In the anion, the bond lengths are P1—O1 = 1.5531 (16), P1—O4 = 1.5638 (17), P1—O2 = 1.5017 (12) and P1—O3 = 1.5024 (14) Å. These values are in full agreement with those found in such anions in other organic dihydrogenomonophosphates [Gholivand *et al.*, 2007; Mrad *et al.*, 2009]. The phosphorus atom has a slightly distorted tetrahedral coordination.

In the crystal structure, the cation and anion components are connected by intermolecular N—H···O and O—H···O hydrogen bonds (Table 1, Fig. 2), with π-π stacking interactions between neighbouring 1,3-thiazole and benzene rings [*Cg*1···*Cg*2*^v^*= 3.5711 (11) Å; symmetry code: (*v*) *x*, 1/2 - *y*, 1/2 + *z*; *Cg*1 and *Cg*2 are the centroids of the S1/N1/C1/C6/C7 1,3-thiazole and C1–C6 benzene rings, repectively], forming a three-dimensional supramolecular network.

### Experimental

The title compound was synthesized using the method of Thomas *et al.* (2003), but with few modifications as follows. 0.1 mole of aniline and 9 ml of concentrated hydrochloric acid were taken in a round bottom flask equipped with reflux condenser, as the insoluble white precipitates of aniline hydrochloride were formed, 25 ml of distilled water was added and the reactants were heated for 35 min. After cooling at room temperature 0.1 moles of sodium thiocyanate were added and the mixture was further refluxed and stirred for 5 h. The resulting mixture was then cooled at room temperature and off-white crystalline solid of phenylthiourea separated out.

0.07 moles of phenyl thiourea were dissolved in 70 ml chloroform in a three-necked round bottom flask equipped with reflux condenser, the whole apparatus was fitted in an ice bath. 0.07 *M* of bromine in 70 ml of chloroform was added drop wise in a period of 2 h in the reaction mixture. The temperature was maintained at 277 K. After the addition of bromine, the mixture was stirred at room temperature for 4 h and was further refluxed for about 3 h until the evolution of hydrogen bromide stopped. On moderate cooling solid separated out filtered and washed with sulfur dioxide water (10 ml conc. H_2_SO_4_ in 50 ml water). The filtrate was neutralized with aqueous ammonia (25%). Precipitates of 2-aminobenzothiazole separated out. Filtered and washed thoroughly with water and re-crystallized in ethanol.

Then 0.001 moles of 2-aminobenzothiazole in 3 ml of methanol and 1–2 drops of *o*-phosphoric acid were added in a round bottom flask. The reaction mixture was refluxed for 8–10 h with continuous stirring. On gradual cooling crystalline solid separated out. Filtered and washed the solid with water and recrystallized in methanol to yield the final product.

### Refinement

Hydroxyl H atoms and H atoms on N atoms were located in a difference Fourier map and refined as riding in their as-found relative positions, with *U*_iso_(H) = 1.5*U*_eq_(O) and *U*_iso_(H) = 1.2*U*_eq_(N). The distances O—H and N—H were restrained to 0.83 and 0.86 Å, respectively. H atoms bonded to C atoms were positioned geometrically and refined using a riding model, [C—–H = 0.93 Å and *U*_iso_(H) = 1.2*U*_eq_(C)].

### Crystal data

**Table:**

C_7_H_7_N_2_S^+^·H_2_PO_4_^−^	*F*(000) = 512
*M**_r_* = 248.20	*D*_x_ = 1.622 Mg m^−^^3^
Monoclinic, *P*2_1_/*c*	Mo *K*α radiation, λ = 0.71073 Å
Hall symbol: -P 2ybc	Cell parameters from 2983 reflections
*a* = 12.3915 (4) Å	θ = 2.6–27.1°
*b* = 10.1572 (3) Å	µ = 0.47 mm^−^^1^
*c* = 8.3159 (2) Å	*T* = 296 K
β = 103.775 (1)°	Rod, off-white
*V* = 1016.56 (5) Å^3^	0.25 × 0.09 × 0.07 mm
*Z* = 4	

### Data collection

**Table:**

Bruker APEXII CCD diffractometer	1938 reflections with *I* > 2σ(*I*)
Radiation source: sealed tube	*R*_int_ = 0.033
graphite	θ_max_ = 28.3°, θ_min_ = 3.2°
φ and ω scans	*h* = −13→16
9333 measured reflections	*k* = −8→13
2490 independent reflections	*l* = −11→8

### Refinement

**Table:**

Refinement on *F*^2^	Primary atom site location: structure-invariant direct methods
Least-squares matrix: full	Secondary atom site location: difference Fourier map
*R*[*F*^2^ > 2σ(*F*^2^)] = 0.038	Hydrogen site location: inferred from neighbouring sites
*wR*(*F*^2^) = 0.096	H atoms treated by a mixture of independent and constrained refinement
*S* = 1.03	*w* = 1/[σ^2^(*F*_o_^2^) + (0.0508*P*)^2^ + 0.1064*P*] where *P* = (*F*_o_^2^ + 2*F*_c_^2^)/3
2490 reflections	(Δ/σ)_max_ = 0.001
151 parameters	Δρ_max_ = 0.31 e Å^−^^3^
5 restraints	Δρ_min_ = −0.25 e Å^−^^3^

### Special details

**Table:**

Geometry. Bond distances, angles *etc*. have been calculated using the rounded fractional coordinates. All su's are estimated from the variances of the (full) variance-covariance matrix. The cell e.s.d.'s are taken into account in the estimation of distances, angles and torsion angles
Refinement. Refinement on *F*^2^ for ALL reflections except those flagged by the user for potential systematic errors. Weighted *R*-factors *wR* and all goodnesses of fit *S* are based on *F*^2^, conventional *R*-factors *R* are based on *F*, with *F* set to zero for negative *F*^2^. The observed criterion of *F*^2^ > σ(*F*^2^) is used only for calculating -*R*-factor-obs *etc*. and is not relevant to the choice of reflections for refinement. *R*-factors based on *F*^2^ are statistically about twice as large as those based on *F*, and *R*-factors based on ALL data will be even larger.

### Fractional atomic coordinates and isotropic or equivalent isotropic displacement parameters (Å^2^)

**Table:**

	*x*	*y*	*z*	*U*_iso_*/*U*_eq_	
S1	0.35481 (4)	0.35955 (5)	1.06135 (6)	0.0400 (2)	
N1	0.28028 (14)	0.13298 (14)	0.95775 (19)	0.0362 (5)	
N2	0.43880 (16)	0.12254 (17)	1.1731 (2)	0.0460 (6)	
C1	0.23286 (16)	0.35055 (18)	0.9039 (2)	0.0363 (6)	
C2	0.16650 (19)	0.4518 (2)	0.8240 (3)	0.0494 (7)	
C3	0.07398 (19)	0.4193 (2)	0.7023 (3)	0.0555 (8)	
C4	0.04759 (19)	0.2898 (2)	0.6589 (3)	0.0525 (7)	
C5	0.11376 (17)	0.1882 (2)	0.7386 (2)	0.0436 (6)	
C6	0.20561 (15)	0.22026 (17)	0.8625 (2)	0.0340 (6)	
C7	0.36161 (16)	0.18903 (19)	1.0689 (2)	0.0350 (5)	
P1	0.31052 (4)	0.78446 (4)	0.06814 (5)	0.0321 (2)	
O1	0.21014 (12)	0.72357 (14)	0.12333 (17)	0.0448 (5)	
O2	0.26558 (12)	0.87196 (11)	−0.07803 (14)	0.0396 (4)	
O3	0.38850 (12)	0.84982 (13)	0.21213 (15)	0.0441 (4)	
O4	0.37402 (14)	0.66563 (15)	0.01419 (16)	0.0497 (5)	
H1	0.271 (2)	0.0479 (16)	0.948 (3)	0.0600*	
H2	0.18390	0.53930	0.85170	0.0590*	
H3	0.02810	0.48600	0.64790	0.0670*	
H4	−0.01510	0.27070	0.57560	0.0630*	
H5	0.09680	0.10090	0.70950	0.0520*	
H6	0.4904 (18)	0.164 (2)	1.242 (3)	0.0600*	
H7	0.430 (2)	0.0391 (17)	1.184 (3)	0.0600*	
H8	0.228 (2)	0.690 (3)	0.214 (2)	0.0750*	
H9	0.379 (2)	0.663 (3)	−0.080 (2)	0.0750*	

### Atomic displacement parameters (Å^2^)

**Table:**

	*U*^11^	*U*^22^	*U*^33^	*U*^12^	*U*^13^	*U*^23^
S1	0.0424 (3)	0.0331 (3)	0.0412 (3)	−0.0075 (2)	0.0035 (2)	−0.0034 (2)
N1	0.0421 (9)	0.0296 (8)	0.0326 (8)	−0.0059 (7)	0.0007 (7)	0.0004 (6)
N2	0.0446 (10)	0.0425 (9)	0.0417 (10)	−0.0074 (8)	−0.0079 (8)	0.0031 (8)
C1	0.0357 (10)	0.0384 (10)	0.0352 (9)	−0.0013 (8)	0.0092 (8)	−0.0010 (8)
C2	0.0530 (13)	0.0418 (11)	0.0525 (12)	0.0090 (10)	0.0110 (10)	0.0023 (10)
C3	0.0512 (13)	0.0605 (14)	0.0515 (13)	0.0172 (11)	0.0055 (10)	0.0062 (11)
C4	0.0370 (11)	0.0749 (16)	0.0409 (11)	0.0027 (11)	0.0000 (9)	0.0000 (10)
C5	0.0400 (11)	0.0500 (11)	0.0383 (10)	−0.0081 (9)	0.0043 (8)	−0.0034 (9)
C6	0.0347 (10)	0.0382 (10)	0.0290 (9)	−0.0026 (8)	0.0074 (7)	0.0013 (7)
C7	0.0371 (10)	0.0350 (9)	0.0321 (9)	−0.0055 (8)	0.0068 (8)	0.0006 (7)
P1	0.0371 (3)	0.0314 (3)	0.0236 (2)	0.0020 (2)	−0.0009 (2)	0.0029 (2)
O1	0.0399 (8)	0.0585 (9)	0.0308 (7)	−0.0073 (7)	−0.0021 (6)	0.0036 (6)
O2	0.0562 (9)	0.0307 (6)	0.0256 (6)	0.0059 (6)	−0.0027 (6)	0.0010 (5)
O3	0.0462 (8)	0.0503 (8)	0.0292 (7)	−0.0127 (6)	−0.0038 (6)	0.0042 (6)
O4	0.0685 (10)	0.0476 (8)	0.0334 (7)	0.0263 (7)	0.0128 (7)	0.0122 (7)

### Geometric parameters (Å, °)

**Table:**

S1—C1	1.7504 (19)	N2—H7	0.862 (18)
S1—C7	1.735 (2)	N2—H6	0.86 (2)
P1—O3	1.5024 (14)	C1—C6	1.389 (3)
P1—O1	1.5531 (16)	C1—C2	1.384 (3)
P1—O2	1.5017 (12)	C2—C3	1.377 (3)
P1—O4	1.5638 (17)	C3—C4	1.382 (3)
O1—H8	0.809 (19)	C4—C5	1.385 (3)
O4—H9	0.801 (17)	C5—C6	1.380 (3)
N1—C6	1.386 (2)	C2—H2	0.9300
N1—C7	1.323 (2)	C3—H3	0.9300
N2—C7	1.314 (3)	C4—H4	0.9300
N1—H1	0.873 (16)	C5—H5	0.9300
			
S1···O4^i^	3.1497 (16)	C1···C7^xi^	3.547 (3)
S1···N1	2.5519 (16)	C1···C5^iii^	3.469 (3)
S1···O3^ii^	3.2897 (15)	C4···C6^xi^	3.496 (3)
S1···C5^iii^	3.664 (2)	C5···S1^xi^	3.664 (2)
S1···C6^iii^	3.5429 (18)	C5···C1^xi^	3.469 (3)
P1···O2^iv^	3.5032 (13)	C6···C4^iii^	3.496 (3)
P1···H6^v^	2.87 (2)	C6···C7^xi^	3.577 (3)
P1···H8^vi^	2.890 (16)	C6···S1^xi^	3.5429 (18)
P1···H9^iv^	2.896 (17)	C7···C6^iii^	3.577 (3)
P1···H1^vii^	2.857 (17)	C7···C1^iii^	3.547 (3)
P1···H7^vii^	3.02 (2)	C3···H3^xii^	3.0400
O1···O2^iv^	2.6017 (18)	C3···H5^xiii^	3.0300
O2···N1^vii^	2.6693 (18)	C5···H3^xiv^	3.0000
O2···P1^vi^	3.5032 (13)	H1···P1^x^	2.857 (17)
O2···O1^vi^	2.6017 (18)	H1···O2^x^	1.800 (16)
O3···N2^vii^	2.875 (2)	H1···H7	2.43 (3)
O3···S1^v^	3.2897 (15)	H2···O4^i^	2.7400
O3···N2^v^	3.138 (2)	H2···O1^i^	2.8900
O3···O4^iv^	2.5654 (18)	H3···C5^xiii^	3.0000
O4···S1^viii^	3.1497 (16)	H3···H3^xii^	2.4100
O4···O3^vi^	2.5654 (18)	H3···H5^xiii^	2.4600
O4···N2^v^	3.076 (2)	H3···C3^xii^	3.0400
O1···H4^ix^	2.6300	H4···O1^xv^	2.6300
O1···H2^viii^	2.8900	H5···H3^xiv^	2.4600
O2···H8^vi^	1.795 (18)	H5···C3^xiv^	3.0300
O2···H1^vii^	1.799 (16)	H6···O4^ii^	2.31 (2)
O3···H7^vii^	2.018 (18)	H6···O3^ii^	2.39 (2)
O3···H9^iv^	1.765 (17)	H6···P1^ii^	2.87 (2)
O3···H6^v^	2.39 (2)	H7···O3^x^	2.018 (18)
O4···H6^v^	2.31 (2)	H7···H1	2.43 (3)
O4···H2^viii^	2.7400	H7···P1^x^	3.02 (2)
N1···O2^x^	2.6693 (18)	H8···P1^iv^	2.890 (16)
N1···S1	2.5519 (16)	H8···O2^iv^	1.795 (19)
N2···O3^ii^	3.138 (2)	H9···P1^vi^	2.896 (17)
N2···O4^ii^	3.076 (2)	H9···O3^vi^	1.765 (17)
N2···O3^x^	2.875 (2)		
			
C1—S1—C7	90.04 (9)	C1—C2—C3	118.06 (19)
O2—P1—O4	109.85 (7)	C2—C3—C4	121.6 (2)
O3—P1—O4	107.32 (8)	C3—C4—C5	120.5 (2)
O1—P1—O2	107.83 (8)	C4—C5—C6	118.07 (19)
O1—P1—O3	110.41 (8)	C1—C6—C5	121.24 (17)
O1—P1—O4	105.70 (8)	N1—C6—C1	112.22 (15)
O2—P1—O3	115.33 (7)	N1—C6—C5	126.55 (17)
P1—O1—H8	112.5 (18)	N1—C7—N2	123.59 (18)
P1—O4—H9	118 (2)	S1—C7—N1	112.46 (13)
C6—N1—C7	114.70 (15)	S1—C7—N2	123.95 (15)
C7—N1—H1	123.5 (16)	C3—C2—H2	121.00
C6—N1—H1	121.7 (16)	C1—C2—H2	121.00
H6—N2—H7	120 (2)	C4—C3—H3	119.00
C7—N2—H6	119.8 (14)	C2—C3—H3	119.00
C7—N2—H7	119.0 (17)	C3—C4—H4	120.00
C2—C1—C6	120.50 (18)	C5—C4—H4	120.00
S1—C1—C6	110.54 (13)	C4—C5—H5	121.00
S1—C1—C2	128.96 (15)	C6—C5—H5	121.00
			
C7—S1—C1—C2	178.1 (2)	S1—C1—C2—C3	179.72 (17)
C7—S1—C1—C6	−1.63 (15)	C6—C1—C2—C3	−0.5 (3)
C1—S1—C7—N2	−178.14 (18)	C2—C1—C6—N1	−178.75 (18)
C1—S1—C7—N1	1.89 (15)	C2—C1—C6—C5	1.6 (3)
C6—N1—C7—N2	178.35 (18)	C1—C2—C3—C4	−0.5 (4)
C7—N1—C6—C5	−179.97 (19)	C2—C3—C4—C5	0.6 (4)
C7—N1—C6—C1	0.4 (2)	C3—C4—C5—C6	0.5 (3)
C6—N1—C7—S1	−1.7 (2)	C4—C5—C6—C1	−1.5 (3)
S1—C1—C6—N1	1.0 (2)	C4—C5—C6—N1	178.86 (19)
S1—C1—C6—C5	−178.61 (15)		

Symmetry codes: (i) *x*, *y*, *z*+1; (ii) −*x*+1, *y*−1/2, −*z*+3/2; (iii) *x*, −*y*+1/2, *z*+1/2; (iv) *x*, −*y*+3/2, *z*+1/2; (v) −*x*+1, *y*+1/2, −*z*+3/2; (vi) *x*, −*y*+3/2, *z*−1/2; (vii) *x*, *y*+1, *z*−1; (viii) *x*, *y*, *z*−1; (ix) −*x*, *y*+1/2, −*z*+1/2; (x) *x*, *y*−1, *z*+1; (xi) *x*, −*y*+1/2, *z*−1/2; (xii) −*x*, −*y*+1, −*z*+1; (xiii) −*x*, *y*+1/2, −*z*+3/2; (xiv) −*x*, *y*−1/2, −*z*+3/2; (xv) −*x*, *y*−1/2, −*z*+1/2.

### Hydrogen-bond geometry (Å, °)

**Table:**

*D*—H···*A*	*D*—H	H···*A*	*D*···*A*	*D*—H···*A*
N1—H1···O2^x^	0.873 (16)	1.800 (16)	2.6693 (18)	174 (3)
N2—H6···O3^ii^	0.86 (2)	2.39 (2)	3.138 (2)	147 (2)
N2—H6···O4^ii^	0.86 (2)	2.31 (2)	3.076 (2)	149.3 (19)
N2—H7···O3^x^	0.862 (18)	2.018 (18)	2.875 (2)	172 (2)
O1—H8···O2^iv^	0.809 (19)	1.795 (19)	2.6017 (18)	175 (3)
O4—H9···O3^vi^	0.801 (17)	1.765 (17)	2.5654 (18)	178 (3)

Symmetry codes: (x) *x*, *y*−1, *z*+1; (ii) −*x*+1, *y*−1/2, −*z*+3/2; (iv) *x*, −*y*+3/2, *z*+1/2; (vi) *x*, −*y*+3/2, *z*−1/2.
